# Neuronal Activity at Synapse Resolution: Reporters and Effectors for Synaptic Neuroscience

**DOI:** 10.3389/fnmol.2020.572312

**Published:** 2020-10-21

**Authors:** Francesco Gobbo, Antonino Cattaneo

**Affiliations:** ^1^Bio@SNS Laboratory of Biology, Scuola Normale Superiore, Pisa, Italy; ^2^Centre for Discovery Brain Sciences, University of Edinburgh, Edinburgh, United Kingdom

**Keywords:** synapse, imaging, synaptic engram, synaptic activity indicators, synaptic activity reporter

## Abstract

The development of methods for the activity-dependent tagging of neurons enabled a new way to tackle the problem of engram identification at the cellular level, giving rise to groundbreaking findings in the field of memory studies. However, the resolution of activity-dependent tagging remains limited to the whole-cell level. Notably, events taking place at the synapse level play a critical role in the establishment of new memories, and strong experimental evidence shows that learning and synaptic plasticity are tightly linked. Here, we provide a comprehensive review of the currently available techniques that enable to identify and track the neuronal activity with synaptic spatial resolution. We also present recent technologies that allow to selectively interfere with specific subsets of synapses. Lastly, we discuss how these technologies can be applied to the study of learning and memory.

## Introduction

Synapses are the physical locus where information is transmitted between neurons. Synapses have specific molecular properties that modulate their effectiveness in propagating neuronal transmission in response to past activity, which confers them a central place in the neurobiology of learning and memory. Of the approximately 30,000 synapses on a single CA1 neuron, the activation of a few hundreds is probably sufficient to activate the cell and initiate action potentials (Routtenberg, 2015). Hence, specific ensembles of synapses could represent unitary bits of information, such as a definite area of space or an element of a memory trace. Which synapses out of the many thousands are responsible for representing these memory elements? Electrophysiological recordings from whole neurons can identify pathways of activation, but lack the ability of creating a map of individual representations at the synaptic level.

Bi-directional modifications of synaptic strength, collectively referred to as synaptic plasticity, have long been regarded as the neural correlate of learning. For instance, the experience-dependent increase of synaptic responses (such as long-term potentiation) is generally believed to be responsible for the behavioral responses observed in associate learning (Martin and Morris, 2002). While not all changes associated to learning and memory involve changes in synaptic transmission (Poo et al., 2016), a comprehensive of the theory of memory would be incomplete without taking into account the role of synaptic changes (Langille and Brown, 2018; Sossin, 2018; Williams and Kyrke-Smith, 2018). Synaptic engrams, the ensemble of synapses undergoing plasticity during learning, represent a parallel trace to cellular engrams. Synaptic engrams mediate the formation of cell assemblies and the probability of their reactivation (Josselyn et al., 2015; Ryan et al., 2015; Lisman et al., 2018), and their role is likely to be more evident in non-associative forms of memories, such as episodic-like memories involving spatial and temporal information (Morris, 2006). How this set of synapses interacts with cellular engrams, and how engram cells are interconnected, is largely still unclear (Poo et al., 2016). For instance, it has been shown that individual memories maintain separated synaptic representations even when they largely overlap at the cellular level (Abdou et al., 2018). Ensembles of active or plastic synapses need not be the lifelong locus of memory storage to be considered candidate synaptic engrams, since engrams are not static entities, but they can evolve over time (Dudai, 2011; Poo et al., 2016). Hence, synaptic engrams can be crucial for memory formation and encoding by mediating the temporal evolution of the engram circuit, even if the original synaptic trace is gradually lost because of protein turnover and spine loss (Mongillo et al., 2017).

To demonstrate the causal relevance of any physiological processes in learning, two conditions have to be met: (i) they need to be identified and take place during learning and (ii) interfering with such process, either positively or negatively, should have a corresponding effect on the ability to form memories (Martin et al., 2000; Tonegawa et al., 2015a). Experiments inducing depression (long-term depression, LTD) or potentiation (long-term potentiation, LTP) of synapses response suggest that altering the synapse status also impacts memory recall (Nabavi et al., 2014), although experiments with synaptic resolutions are still lacking. When it comes to the specific sets of synapses activated by experience, testing the two criteria has long been an experimental challenge, due to the lack of suitable techniques to identify and manipulate active synapses in a reliable manner (Takeuchi et al., 2014). In recent years, however, a number of techniques have been described to map and intervene on ensembles of synapses, based on their activity. Here, we present the currently available technologies and critically discuss how these could be applied to the study of memory at the synaptic level (Table 1).

**Table 1:** List of the main currently available sensors and effectors for synaptic activity.

Indicator	Brief description	Type	Applications	Expression	Source	
*Genetic*
Actin-GCaMP2	Calcium imaging reporter fused to beta actin. Enriched at spines	Activity	*In vivo* live imaging	Constitutive	Addgene 18928
GCaMP3	Calcium imaging reporter	Activity	*In vivo* live imaging. Applied to image *Drosophila* presynaptic boutons activity. Tuft dendritic potentials in mice	Constitutive	Tian et al. (2009) Addgene 43917, 32644
GCaMP6s	Calcium imaging reporter with sufficient SNR to detect synaptic events	Activity	*In vivo* live imaging. Cortical areas, deeper regions with chronic windows (hippocampus). Pre- and postsynaptic structures. Extensively tested in rodents, zebrafish and *Drosophila*	Constitutive and floxed forms. Suitable for AAV delivery and *in utero* electroporation. Transgenic animals available	Chen et al. (2013) Addgene 40753, 100842 (AAV vector), and others Transgenic mouse lines available: Camk2a-tTA (B6.Cg-Tg(Camk2a-tTA)1Mmay/DboJ, Jax #007004, RRID:IMSR_JAX: 007004, and others
jGCaMP7b	Calcium imaging reporter with sufficient SNR to detect synaptic events	Activity	*In vivo* live imaging. Cortical areas, deeper regions possible with chronic windows (hippocampus). Pre- and postsynaptic structures (mostly rodents)	Constitutive and floxed forms. Suitable for AAV delivery and, likely, *in utero* electroporation	Dana et al. (2019) Addgene 104489, 104497 Addgene 135419 (variant enriched at axons)
jRECO1a	Red fluorescent calcium imaging reporter with sufficient SNR to detect synaptic events	Activity	*In vivo* live imaging. Cortical areas, such as visual cortex. Tested in rodents, *Drosophila*, and zebrafish	Constitutive for AAV delivery. Floxed versions available. Other methods possible	Addgene 100852, 100853, 100854 *Drosophila* strain PBac20 × UAS-IVS-NES-jRCaMP1a-p10VK00005 Flybase ID FBti0180188
XCaMP series: XCaMP-Y XCaMP-R	Multicolor variants of calcium imaging reporters with sufficient SNR to detect synaptic events	Activity	*In vivo* live imaging. Cortical areas, such as visual cortex. Imaging in the hippocampus is possible with longer wavelength sensors	Constitutive	Inoue et al. (2019)
Syntagma	Photoconvertible CAMPARI variant (green to red). The photoconversion requires calcium influx and UV light (*via* either an optic cannula or imaging objective)	Activity	*In vivo* live imaging. Fixed tissue. Pre- (PSD95.FingR fusion) and postsynaptic (synaptophysin fusion) variants available. Tested in rodents	Constitutive. AAV delivery	Perez-Alvarez et al. (2020) Addgene 119738 (pre) Addgene 119736 (post)
SF-iGluSnFR A184S (higher SNR) or S72A (higher temporal fidelity) variants	Fluorescent reporter for glutamate release	Activity	*In vivo* live imaging. Cortical areas, tested in the rodent and ferret visual and sensory cortex	Constitutive. AAV delivery	Marvin et al. (2018) Addgene 106200 (S72V variant), Addgene 106198 (A184S variant)
SEP-GluA1	AMPA receptor trafficking and exposure	Plasticity (E-LTP)	*In vivo* live imaging	Constitutive. *In utero* electroporation. Knock-in mouse line reported	Addgene 24000, Addgene 64942 Transgenic line: Graves et al. (2020)
GFP-GluA1	AMPA receptor trafficking and exposure	Plasticity (E-LTP)	*In vivo* imaging. Fixed tissue	Constitutive and inducible	Addgene 34857
AS-PaRac1	Local translation of light-sensitive Rac1	Plasticity (L-LTP)	*In vivo* live imaging. Fixed tissue	Constitutive or activity dependent. AAV delivery and *In utero* electroporation reported	Hayashi-Takagi et al. (2015)
SA-Ch	Local translation of ChR2 variant	Plasticity (L-LTP)	Live
imaging. Fixed tissue	Inducible. *In utero* electroporation	Gobbo et al. (2017)
Diffusible fillers	Fluorescent fillers of the GFP and RFP families can be used to image changes in dimensions and new spine formation or elimination. Membrane-anchored fluorescent proteins can also be used to increase signal	Structural plasticity (changes in dimension and formation/removal of spines)	Live imaging. Validated in multiple models including rodents, *Drosophila*, zebrafish	Any method of delivery. Multiple transgenic lines are available	Multiple commercial sources. Transgenic lines: B6.Cg-Tg(Thy1-YFP)HJrs/J Jax #003782 and many others
PSD-95 fluorescent variants (or Homer1c fusions)	Fluorescent postsynaptic proteins (excitatory synapses)	Structural plasticity (mainly formation/loss)	Live imaging	Multiple methods of delivery. Transient or inducible expression may be preferable	Addgene 125694, 125693, 133785, and others
Gephyrin fluorescent variants	Fluorescent postsynaptic proteins (inhibitory synapses)	Structural plasticity (mainly formation/loss)	Live imaging	Multiple methods of delivery. Transient or inducible expression may be preferable	Addgene 126217, 73918, and others
e-GRASP	Complementation system to visualize synapses between defined pre- and postsynaptic neuron populations. It can be activity dependent. It can be applied to inhibitory connections (test needed)	Structural plasticity (changes in dimensions and numbers), plasticity of connections	Live imaging possible. Fixed tissue	Constitutive *via* AAV delivery. Activity-dependent expression is dependent on the tetON system (cfos-tTA)	Addgene 111579 (cyan pre-eGRASP) 111582 (tet-dependent yellow pre-eGRASP), 111580 (yellow pre-eGRASP), 111584 (post-eGRASP) Addgene 120309 (cfos-tTA)
*Chemical*
FM dyes	Neurotransmitter exocytosis	Activity	Live imaging	
Injected	Commercial sources available
Oregon Green 488 BAPTA-1, Fluo-4 (and others)	Pre- or postsynaptic activity	Activity	*In vivo* live imaging	Injected, delivered with patch pipettes or cell electroporation	Commercial source available

*We indicate their main use and commercial course, if available. Some plasmids and all chemicals listed can be purchased from a number of specialized for-profit commercial companies. Where the tool is particularly diffuse, we indicate some example sources that reflect the original or most diffuse forms (or transgenic lines). Abbreviations: AAV, adeno-associated vector; GFP, green fluorescent protein; RFP, red fluorescent protein; SNR, signal-to-noise ratio*.

## Tagging Synaptic Engrams: Comparison With Cellular Engrams

The identification of immediate early genes (IEGs), genes that are rapidly transcribed when neurons are electrically activated (Yap and Greenberg, 2018), opened the way to the definition of cellular engrams, i.e., populations of neurons that are believed to embody specific memory traces in the brain (Sakaguchi and Hayashi, 2012; Mayford, 2014). In these experiments, neurons activated during a certain behavioral paradigm are genetically tagged by driving the expression of the desired transgene from an exogenous transcription factor (TF) under an IEG promoter (usually, c-fos). This TF is sensitive to drugs that can be delivered to the animal and can restrict the expression of the transgene, depending on their availability (Reijmers et al., 2007; Ramirez et al., 2013). If opto- or chemogenetic proteins are expressed during the learning phase, the role of this neuronal population can be tested by re-activating them at wish or silencing them during natural recall. The results by the Mayford, Tonegawa, and Josselyn groups support the idea that the ensemble of neurons that participate in learning are also involved in the retrieval of a memory (Josselyn and Tonegawa, 2020). In these experiments, neurons are regarded as unitary entities: all neurons, whose activity exceeds a given threshold in the time window controlled by the drug administration, will be tagged, and the transgene will be expressed in the whole cell. Later reactivation causes tagged neurons to fire at the same level, thus losing information on the subcellular distribution of inputs or input specificity. It is remarkable that such reactivation paradigms are sufficient to elicit behavioral responses consistent with the encoded memory, even if so far the majority of behavioral paradigms used rely on contextual fear conditioning (Liu et al., 2012) and place preference (Ramirez et al., 2013), and sometimes only some stimulation protocols are effective (Ryan et al., 2015).

The engram cell theory, which regards engram cells as the unitary elements of memory allocation and storage, is not informative on the possibility that unitary elements of memory are represented at the subcellular level. Given the complexity of the neuronal inputs, it is possible that ensembles of individual synapses (Sakaguchi and Hayashi, 2012; Dudai and Morris, 2013; Nomoto and Inokuchi, 2018)—or of clustered groups of synapses (Kastellakis et al., 2015)—contribute to the initial representation of a memory, at least temporarily, to its storage in the form of a synaptic engram. Furthermore, little is known about how neurons are recruited into cellular engrams, nor which synaptic connections are relevant in their parallel or hierarchical activation. Studies of learning-related plasticity show that plasticity is synapse-specific, i.e., it can occur at some but not all synapses made by an individual neuron. Multiple engrams can also co-exist as separate traces even if these share a subset of neurons (Abdou et al., 2018). Thus, synaptic engrams may exist embedded within cellular engrams, and synaptic connections between individual engram cells in an engram cell assembly can be stabilized differently. Even if it is possible that the ultimate site of memory store resides in a stabilized neural circuit (Tonegawa et al., 2015b), there is strong evidence that active synapses during learning are necessary for the initial storage of a memory and for its later recall (Poo et al., 2016).

**Figure 1:**
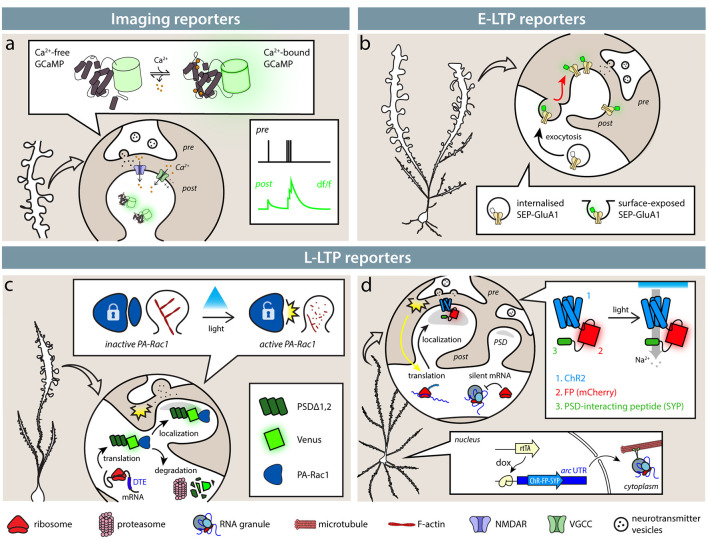
Available tools identify synapses based on their activity. **(A)** Imaging reporters (genetically encoded calcium indicators, GECIs), such as calcium (Ca^2+^) indicators, allow the experimenter to identify active synapses in live imaging. Ca^2+^ influx through NMDARs (blue) and VGCCs (green), as well as from intracellular stores, causes GECIs, such as GCaMP6s, to shift from a dark to a fluorescent state. Synaptic activity then results in an instantaneous change in fluorescence. **(B)** Exposure of SEP-tagged AMPA receptors (SEP-GluA1) labels synapses during E-LTP. In intracellular stores, SEP fluorescence is quenched by the acidic pH, so that only exposed AMPA receptors following synaptic activity are fluorescent. **(C,D)** SA-Ch and AS-PaRac1 reporters are expressed at synapses following potentiation induction. **(C)** AS-PaRac1 is expressed at synapses following potentiation thanks to Arc dendritic targeting element (DTE) in the 3′-UTR. PSDΔ1, 2 anchors the protein to the postsynaptic density (PSD) and promotes its degradation outside the synapse. AS-PaRac1 encodes a light-sensitive Rac1 form fused to Venus fluorescent protein. When it activated by blue light, it causes actin depolymerization and spine shrinkage. **(D)** SA-Ch encodes the ChR2 variant ChETA fused to the red fluorescent protein (RFP) mCherry and to SYP tag interacting with the PSD. Arc UTR sequences maintain the mRNA in repressed state and allow its translation at potentiated synapses. Like ChR2, SA-Ch is a cation channel that causes depolarizing photocurrents, but in principle, it could be substituted by opsins of other ionic specificities.

We can therefore ask whether synaptic engrams can be defined using similar operational criteria that have been used to define the cell engrams, i.e., synapses activated and modified by a learning experience, whose reactivation is necessary and sufficient to support memory retrieval. One major difficulty in the identification of synaptic engrams is the need to develop experimental techniques to map and act on these synaptic ensembles, based on the activity-dependent long-lasting modifications they undergo during a learning process. At the neuron level, IEGs expression proved to be a reasonable and robust hallmark to identify candidate cellular engrams. Indeed, IEGs have a series of sound properties for an engram candidate, such as being expressed at the time of memory formation (Guzowski et al., 1999) and being intrinsically related to synaptic and cellular plasticity (Gallo et al., 2018; Yap and Greenberg, 2018). Moving from the whole cell to the single-synapse level feels much like moving into a *terra incognita*, where many of the molecular events taking place in the active synapse are known, but a comprehensive picture of their relative importance is still missing. Nevertheless, for a set of synapses to form a memory-relevant trace, they should be active during the learning phase—even though silent synapses (Wang et al., 2019) and newly formed synapses may also play a role (Xu et al., 2009; Yang et al., 2009). As we shall see in the next sessions, a number of molecular events that follow synaptic activation have been used to develop reporters to identify active synapses in learning tasks (Figure 1).

## Imaging Synaptic Activity

One obvious possibility to identify active synapses is to image their activity with single-synapse resolution. The introduction of reporters of neural activity with sufficient sensitivity to measure synaptic events made it possible to extend the analysis to single-synapse resolution. A now widely diffuse class of reporters of synaptic activity is represented by genetically-encoded calcium indicators (GECIs): now almost two-decades old, circularly permutated green fluorescent protein (cpEGFP)-based GCaMP series is based on the insertion of a calmodulin and a calmodulin-binding peptide (M13) within the EGFP protein sequence (Nakai et al., 2001; Figure 1A). Calcium binding forces calmodulin to assume a more compact shape, causing a net increase of EGFP fluorescence. Calcium is a potent signaling ion for synaptic activity: the influx of calcium ions at the presynaptic site is responsible for neurotransmitter release (Südhof, 2012), whereas at the postsynaptic site, Ca^2+^ ions can flow in through NMDA receptor (NMDAR) and Ca^2+^-selective channels (Higley and Sabatini, 2012).

The first successful recording of synaptic activity with synaptic resolution was performed fusing GCaMP2 sensor to synaptophysin, making it possible to visualize the incoming action potential at presynaptic terminals (Dreosti et al., 2009). A proof-of-principle observation of synaptic transmission at the postsynaptic site was performed by fusing GCaMP2 to actin, hence enriching the sensor to actin-rich spines (Mao et al., 2008). However, it was not until the development of GECIs with higher sensitivity and intensity that reliable imaging of synaptic activation could be achieved. By expressing GCaMP3 in the somatosensory cortex, Xu et al. (2012) visualized the response of tuft dendrites to whisker sensing. A major improvement was the introduction of GCaMP6s: expressing GCaMP6s in the visual cortex, Chen et al. (2013) were able to detect single synaptic events, showing that single synapses were selectively activated by visual stimuli of different orientations. Orientation preference was stable over at least 27 days of repeated imaging of the same neurons (Chen et al., 2013). The latest class of GECIs has thus proven able to identify synaptic events with submicron resolution, hence in principle allowing the recording of the activity of single synapses across days. To date, the two GECI variants that allow this level of resolution are GCaMP6s (Chen et al., 2013) and jGCaMP7b (Dana et al., 2019). They now exist in multicolor, which could find a useful application when it comes to spectra overlap or cell-type specific analysis (Inoue et al., 2019). For instance, the red indicator jRECO1b has been used in conjunction with GCaMP6s in the visual cortex to visualize the synaptic activity of the synapses on layer 5 neurons and of axons from the lateromedial area simultaneously (Dana et al., 2016). Synthetic calcium indicators have also proven to be reliable tools to image synaptic activity, with performance equal (if not sometimes superior) to GECIs, and have been applied extensively to the imaging of synaptic activity in cortical areas, such as the somatosensory cortex, where spontaneous and evoked responses were detected with two-photon imaging (Varga et al., 2011).

### Significance of Synaptic Activity Imaging

Identifying active synapses during the presentation of a given sensory stimulation is a fundamental first step toward the identification of a synaptic correlate of memory representation and encoding. Stability is generally believed to be a necessary feature for a candidate synaptic representation of a memory; indeed, the constancy of orientation selectivity in the visual cortex seems to meet this requirement. Similar considerations were applied to neurons in other cortical areas using synthetic calcium indicators, such as Oregon-Green Bapta1 or Fluo 5F. In the auditory cortex, single spines in pyramidal neurons can be repeatedly activated by a sound of their preferred tone frequency (Chen et al., 2011), whereas in the barrel cortex, synaptic selectivity has been measured by visualizing synaptic activity in response to single whisker stimulation (Takahashi et al., 2012).

It is not clear, however, what the ensemble of active synapses represents, whether they simply reflect the transmission of information between the imaged neurons, or they actually encode a memory for the specific stimulus. In principle, potentiation of synaptic transmission can be inferred with GECIs from an increase in amplitude of the calcium transients. In hippocampal slices, increases in ΔF/F ratios (the standard measure to express changes in fluorescence with indicators) after optical LTP induction correlate with changes in spine volume and potentiation of synaptic transmission measured in patch-clamp mode (Wiegert et al., 2018). If applied *in vivo*, this could provide an estimate of functional potentiation of synaptic transmission at synapse resolution. By expressing GCaMP at Kenyon cells presynaptic sites onto mushroom bodies in *Drosophila*, Bilz et al. (2020) showed that different odors can be represented at the level of separate synaptic boutons and display input-specific synaptic plasticity during associative learning. Furthermore, the time course of potentiation could be measured too, distinguishing between transient and long-lasting effects; however, it is not clear if all types of plasticity could be measured in this way, and it would be essential to estimate carefully the amplitude and variance of the ΔF/F synaptic events in the calculation of the baseline.

### Technical Considerations

In principle, imaging synaptic activity could provide a large amount of information. This information could be so large that it poses the difficulty of how to interpret and analyze this type of data, extracting consistent patterns out of the multitude of synaptic events. In practice, however, the field of view is limited by spatial and temporal resolution constraints. State-of-the-art imaging with opto-acoustic deflectors (AODs) can reasonably image volumes of around 10^5^ μm^3^, which correspond to a cube of 50 μm per side (Schultz et al., 2016; Verstraelen et al., 2018). Larger volumes can be images using random-access methods, which are based on a prior definition of the sites of interest to be imaged within volumes of around 150 μm per side (Fernández-Alfonso et al., 2014). Similar results are obtained with advanced imaging setups coupling AODs with Bessel laser beams, making it possible to record at 30 Hz from around 5 × 10^6^ μm^3^ (equivalent to 270 × 270 × 60 μm) with submicron resolution (Lu et al., 2017).

Imaging live synaptic activity poses additional experimental limitations. First, it only allows imaging of brain areas that are accessible to two-photon microscopes. In practice, this often translates into cortical areas, where imaging can be performed through optical windows or thinned skull (Chen et al., 2011). This has also precluded imaging during most behavioral paradigms, because two-photon imaging is generally performed on head-fixed animals. In particular, this precludes its application to spatial tasks that are not based on virtual reality (Gauthier and Tank, 2018). Notably, the first record of synaptic Ca^2+^ transients with a head-mounted two-photon microscope has only been reported in 2017 (Zong et al., 2017), after previous attempts suffered technical limitations that prevented effective *in vivo* recording of submicrometric structures, such as spines. Imaging deeper brain areas, such as the hippocampus, at synaptic resolution can be achieved coupling two-photon imaging with GRIN lenses located within the brain to gain access to the desired brain region (Attardo et al., 2015; Meng et al., 2019). It is therefore conceivable to couple the head-mounted two-photon microscopes with GRIN lenses to image synaptic activity in the hippocampus during learning tasks. Technological advances in optical imaging promise to push the limits of temporal (Wu et al., 2020) and spatial resolutions (Lu et al., 2017) to allow live imaging of synaptic activity.

### Other Synaptic Activity Imaging Techniques

cpEGFP-based GECIs are the most common class of calcium sensors employed in neuroscience research. Other classes of GECIs exist, for example, those based on FRET emission between two fluorescent proteins with different emission spectra (Kotlikoff, 2007). Efficient voltage sensors have also been described recently, and while they have been applied to the visualization of the activity of presynaptic terminals in *Drosophila* (Yang et al., 2016), imaging voltage changes at the synaptic resolution probably needs variants with even higher brightness and signal-to-noise ratios (SNRs; Bando et al., 2019). In addition, fluorescent indicators for glutamate release and pH-sensitive dyes for vesicle fusion have been described. In particular, the glutamate sensor iGluSnFR (Marvin et al., 2013) and its variants, which signal glutamate binding by an increase in fluorescence, have shown generally good performance in detecting synaptic events of glutamate release (Jensen et al., 2019), making them a viable alternative to image synaptic activity. Recently, an improved iGluSnFR variant has been employed *in vivo* to visualize orientation selectivity response at individual synapses in the mouse and ferret visual cortex (Marvin et al., 2018). Genetic pH-sensitive fluorescent indicators expressed on the membrane of neurotransmitter vesicles can be used to monitor presynaptic activity when they fuse with the plasma membrane, although to our knowledge, they have so far only been employed in large synapses (neuromuscular junctions, calix of Held; Kavalali and Jorgensen, 2014). Lastly, also sensors to image intracellular signaling events have been developed (e.g., CaMKII and RhoA activation), although their use *in vivo* has been limited. A comprehensive description of these sensors is beyond the scope of this article, and we refer to other excellent reviews (Padamesey and Emptage, 2011; Lee et al., 2016; Lin and Schnitzer, 2016).

Recently, a method to take a “snapshot” of active synapses at a given time has been described (Perez-Alvarez et al., 2020). This technique, named Syntagma, is based on the green fluorescent protein (GFP) CAMPARI that can be photoconverted into a red form with blue-light illumination in the presence of high Ca^2+^ concentrations (Moeyaert et al., 2018). By fusing the CAMPARI fluorescent protein to either presynaptic synaptophysin or a postsynaptic anti-PSD95 intracellular antibody, they were able to provide a snapshot of active synapses during illumination. Computational offline analysis enables the identification of thousands of individual active synapses and the description of their status (active–inactive) from their fluorescence (red–green). This approach frees calcium imaging from the constraints of live recording, thus extending it to non-restrained behavior and enlarging the imaging volume, with the only constraint being the area reached by sufficient light power. Furthermore, any brain area can in principle be analyzed with this technology by using an appropriate optic guide.

While the focus of this review lies in genetic methods, so that the reporters can be expressed as transgenes in the brain using standard molecular genetics techniques, such as adeno-associated vectors (AAVs) or Cre-lox systems (Sjulson et al., 2016; Haery et al., 2019), in some cases, chemical compounds can be used to image synaptic activity *in vivo* or in acute slices. These can usually be delivered in soluble form *via* patch pipettes or similar [e.g., Fluo-4, Fluo-5F, Oregon Green BAPTA-1 (OGB)] or injected in a form that can be taken up by neurons (e.g., OGB-AM). Organic calcium dyes are bright calcium indicators and have been successfully used to monitor synaptic activity *in vivo* (Varga et al., 2011; Winnubst et al., 2015). In the same way, fillers, such as organic fluorophores, can be used to image structural plasticity at synapses (see next section; Fernández-Alfonso et al., 2014). Presynaptic activity can be monitored with organic dyes that accumulate in neurotransmitter vesicles or their membrane (e.g., FM dyes); usually, neural activity results in negative changes in fluorescence due to vesicle fusing with the plasma membrane, thus limiting their use to *ex vivo* preparations (Kavalali and Jorgensen, 2014). In some cases, specific vesicle types (e.g., dopaminergic vesicles) can be labeled with fluorescent neurotransmitter analogues to monitor synaptic activity (Pereira et al., 2016).

## Synaptic Events During Potentiation

From the perspective of synaptic engrams, we must distinguish between synaptic activity and synaptic plasticity mechanisms. Activity at synapses during the learning phase may contribute differently to neuronal representations: while some of the active synapses may be involved in long-term information storage, others may just reflect signal transmission, neuronal computation, or activity noise (Pouget et al., 2013). An appealing candidate for synaptic storage of memories is the ensemble of synapses undergoing potentiation (Mayford et al., 2012; Rogerson et al., 2014; Takeuchi et al., 2014). During a learning process, patterns of neural activity representing the learned events cause changes in the strength of synaptic connections (Whitlock et al., 2006). According to Semon’s engram definition (Schacter et al., 1978), synapses potentiated during the presentation of a stimulus satisfy the requirements for an engram candidate: they are activated by the occurrence of a stimulus, and they undergo modifications that change their response as a consequence of stimulus presentation. On a third point, i.e., if their reactivation can start the memory recall, a definitive answer is still lacking, although there is evidence pointing in its support (Nabavi et al., 2014; Abdou et al., 2018).

*In vivo* potentiation is believed to recapitulate what happens during electrophysiological induction of LTP (Andersen et al., 2017). A cascade of events following glutamatergic stimulation involves a complicated intracellular signaling cascade, including αCaMKII phosphorylation and remodeling of the actin cytoskeleton (Herring and Nicoll, 2016). Two phases can be identified within LTP, namely, early (E-) and late (L-LTP; Mayford et al., 2012). E-LTP is a rapid increase in the efficiency of synaptic transmission and typically lasts a few hours or less, whereas L-LTP can last for several hours (Kelleher et al., 2004; Wang et al., 2010). E- and L-LTP should be regarded as two separate processes taking place at the same time, although they clearly interact with each other (Reymann and Frey, 2007; Bliss et al., 2018). E-LTP induction is not sensitive to translation inhibitors and is generally thought to be dependent on the exposure of more AMPA-sensitive glutamate receptors (AMPARs), whereas L-LTP expression and maintenance is translation dependent (Kelleher et al., 2004). The two forms are believed to have different intensity thresholds for induction, and L-LTP-like forms of potentiation can be even induced in the absence of E-LTP (Bliss et al., 2018), for example, through the activation of the BDNF/TrkB pathway (Kang and Schuman, 1996). Furthermore, multiple forms of LTP can co-exist within the same neuronal population, which may have different requirements in terms of molecular pathways involved (Edelmann et al., 2017).

Potentiation is usually accompanied by structural events, such as the enlargement of pre-existing synapses and the formation of new synapses. Matsuzaki et al. (2004) showed that inducing LTP at specific synapses causes an increase in spine volume, which is accompanied by an analogous increase in the dimension of the postsynaptic density (PSD; Meyer et al., 2014). Similarly, learning is accompanied by new spine formation and stabilization (Xu et al., 2009; Yang et al., 2009). These events are relatively simple to identify and follow over time, by expressing untargeted (Chen et al., 2000), membrane anchored (Cai et al., 2013), or postsynaptic fluorescent proteins that fill the neurons. However, absolute spine dimension or spine emergence is only quite an indirect proxy of the synapse status. Furthermore, spines are typically underresolved by standard imaging techniques—with the exception of super resolution imaging—which can make it harder to evaluate spine dimensions and to distinguish two spines in close proximity (Attardo et al., 2015; Pfeiffer et al., 2018).

## Synaptic Potentiation Reporters: E-LTP

During E-LTP, AMPARs are exocytosed onto the surface of the plasma membrane in proximity to the synapse, where they can diffuse in the lipid bilayer until they are captured and retained in the PSD by interactions with scaffold proteins, such as PSD95 (Chater and Goda, 2014). Initially designed to follow AMPAR mobility, fusion proteins between an AMPAR subunit and fluorescent EGFP later became reporters to identify activated synapses in the brain (Tanaka and Hirano, 2012). The most diffuse of such reporters is a fusion protein between the rat AMPAR subunit 1 (SEP-GluA1) and a pH-sensitive EGFP variant, superecliptic pHluorin (SEP). SEP is inserted at the N-terminus of the AMPAR subunit after the signal peptide, which faces the extracellular side of the plasma membrane. At physiological pH, it displays bright green fluorescence, but it is severely quenched at acidic pH, typical of the endosomal compartment (Kopec et al., 2006). This means that when AMPARs containing subunit 1 are exposed at the surface of the plasma membrane, these sites can be identified by changes in fluorescence.

When expressed *in vivo* in layer 2/3 pyramidal neurons in the barrel cortex, SEP-GluA1 enabled the identification of synapses undergoing potentiation following whisker stimulation (Figure 1B; Makino and Malinow, 2011; Zhang et al., 2015). Two-photon imaging allowed the authors to map the position of potentiated synapses by looking at the accumulation of surface SEP-GluA1, showing that sensory stimulation causes clustering of synaptic upscaling (Makino and Malinow, 2011). The sensory-dependent accumulation of SEP-GluA1 to synapses is dependent on NMDAR activity, since its antagonist CPP prevented the observed change in fluorescence. Notably, the observed increase in surface SEP-GluA1 after whisker stimulation is stable for up to 48 h, and in some cases, SEP-GluA1 intensity could be identified reliably on the same spine for up to 28 days (Zhang et al., 2015).

GFP-tagged AMPAR subunit 1 has been used in conjunction with the Tet-tag system (Reijmers et al., 2007) to image active synapses in hippocampal CA1 neurons, during a time window controlled by doxycycline, of animals exploring a novel context (Takahashi et al., 2012) or undergoing contextual fear conditioning (Matsuo et al., 2008). The GFP-GluA1^+^ synapses are particularly represented in mushroom spines after fear conditioning, which are usually considered more mature spines.

### Significance of AMPAR-Based Reporters

While NMDA receptors are generally necessary for LTP induction in glutamatergic synapses, the recruitment of further AMPA receptors is responsible for the early increase in synaptic currents. Hence, synapses tagged with fluorescent AMPAR subunits are supposed to be the subset of synapses where activity reached the threshold to induce E-LTP. AMPARs containing only GluA1 subunits are preferentially recruited shortly after synaptic activity, whereas GluA2- and GluA3-containing AMPAR incorporation only takes place at later times (Tanaka and Hirano, 2012; Diering and Huganir, 2018). Therefore, SEP-GluA1 containing spines likely represent the pool of synapses that underwent E-LTP—or received an equivalent physiological stimulation—a subset of those of course may also present L-LTP. Even if GluA2/3 containing synapses have been linked to a later stage of memory, such as consolidation and reconsolidation (Diering and Huganir, 2018; Shehata et al., 2018), it would be unclear then what a SEP-GluA2 (or SEP-GluA3) reporter could represent in terms of synaptic population, since SEP-GluA2^+^ synapses do not show analogous clustering to SEP-GluA1^+^ after whisker stimulation (Makino and Malinow, 2011).

While the initial change in surface SEP-GluA1 is positively correlated with the increase in spine size in the first hour, on average, there was no lasting change of spine dimension (Zhang et al., 2015). This suggests that these synapses underwent only a transient increase in synaptic transmission. This does not preclude their identification even when potentiation has decayed: once incorporated in the pool of synaptic AMPARs, SEP-GluA1 molecules could be maintained as a lasting proportion of AMPARs, even when the overall number of AMPARs has returned to pre-stimulation levels. It is unclear what causes the retention of SEP signal even a month after whisker stimulation, since protein degradation likely eliminated SEP-GluA1 molecules that were initially incorporated; it is not clear how ongoing translation could maintain the asymmetric incorporation of new SEP-GluA1 between synapses.

### Technical Considerations

SEP-GluA1 and GFP-GluA1 reporters for (E-)LTP can provide useful information in the identification of activated synapses. The use of SEP fusions rather than the GFP version reduces background from non-exposed AMPARs. However, loss of pH gradients after fixation makes the use of SEP-GluA1 only advantageous for live imaging experiments (Kopec et al., 2006); hence, it is usually limited to *in vivo* imaging of cortical areas through optical windows (Zhang et al., 2015) or acute slices (Makino and Malinow, 2011). *Ex vivo* analysis of fixed samples requires surface immunostaining of SEP/GFP-GluA1, which is facilitated by the generally good performance of anti-GFP antibodies, but is dependent on the fixation and antigen recognition conditions (Matsuo et al., 2008).

Furthermore, the use of SEP/GFP-GluA1 usually suffers from variable background signal, which is mostly due to the passive incorporation of the reporter GluA1 at synapses due to the normal turnover of AMPARs. In many cases, SEP-GluA1 incorporation is therefore expressed as change in intensity rather than absolute fluorescence (Araki et al., 2015; Zhang et al., 2015), which makes the identification of the subset of synapses involved in a given task trickier. In any case, restricting the temporal expression of the reporter, by controlling it with doxycycline or tamoxifen-sensitive Cre systems, can improve specificity and reduce background (Matsuo et al., 2008; Makino and Malinow, 2011).

## Synaptic Potentiation Reporters and Effectors: L-LTP

New translation is necessary for L-LTP (Kelleher et al., 2004), and protein synthesis from dendritically localized transcripts has been shown to be a key event following stimulations inducing L-LTP (Sutton and Schuman, 2006; Holt et al., 2019). Dendritic RNAs serve a number of purposes ranging from providing effector proteins (e.g., Arc, PKMζ, αCaMKII), producing key proteins for LTP expression (e.g., PSD95, GluA1), and supporting homeostatic protein turnover (Cajigas et al., 2012). Transcripts are transported and maintained in the dendrites in a translationally repressed state due to *cis* RNA sequences (elements allocated in the transcript as part of the ribonucleotide sequence) that are bound to molecular motors, regulatory proteins (e.g., FMRP), stalled components of the protein synthesis machine (e.g., CPEB, EJC proteins, PABP), miRNA, and other regulatory RNAs (Fernandez-Moya et al., 2014). A large number of RNAs are present in dendrites, with most of the containing dendritic targeting sequences (DTEs) in their 3′-UTR, specific sequences responsible for transport and regulation (Cajigas et al., 2012). Among them, the RNA of the IEG *arc* has been shown to rapidly and abundantly translocate in the dendritic layer after high-frequency stimulation of the dentate gyrus from the perforant path (PP), accumulating in correspondence to the activated synapses (Steward and Worley, 2001; Dynes and Steward, 2012; de Solis et al., 2017). *Arc* translation is induced by LTP stimuli and has been observed to occur in correspondence to dendritic synapses (Bramham et al., 2010; Minatohara et al., 2016; Na et al., 2016).

Two groups have used the properties conferred by *arc* untranslated regions (UTRs) to engineer protein reporters and actuators to be specifically translated at potentiated synapses (Hayashi-Takagi et al., 2015; Gobbo et al., 2017).

Hayashi-Takagi et al. (2015) generated AS-PARac1, a photoactivable Rac1—thanks to a photosensible LOV domain (Wu et al., 2009)—fused to a fluorescent protein, expressed from a mRNA that bears *Arc* dendritic targeting element (DTE; Kobayashi et al., 2005) in its 3′-UTR (Figure 1C). The DTE sequence conferred activity-dependent translation of the reporter protein, which was anchored at the postsynaptic membrane in the spine by a PSD-binding moiety consisting of a deletion mutant of PSD95 (PSDΔ1,2). AS-PARac1 labeled synapses sparsely when expressed in hippocampal organotypic cultures and in the motor cortex *in vivo*, and following a motor task training, the reporter preferentially labeled enlarged and newly formed spines. Focal LTP induced by glutamate uncaging resulted in the expression of AS-PARac1 at stimulated synapses only, which was dependent on new protein synthesis. Therefore, the AS-PARac1 reporter allows mapping of the potentiated synapses. Notably, the AS-PARac1 reporter also allows intervention on the labeled synapses, since Rac1 is a small GTPase that regulates cytoskeleton organization. In the dark, the Rac1 activity of the AS-PARac1 reporter protein, expressed at potentiated synapses, is inhibited by steric hindrance by the photosensible LOV domain, and a pulsed blue-light stimulation can remove the LOV block (Wu et al., 2009), depolymerizing the actin filaments inside synapses. This resulted in labeled spines to irreversibly collapse and shrink in dimension, and accordingly, synaptic transmission at these potentiated synapses appeared to be severely, if not completely, impaired.

Since AS-PARac1 is a soluble protein, is it possible to express also membrane proteins, such as excitatory or inhibitory optogenetic channels at potentiated synapses? This would allow a functional and reversible modulation of the activity of synapses that had been potentiated by a previous learning activity. Gobbo et al. (2017) addressed this question by driving the expression of the ChR2 Channelrhodopsin variant (Gunaydin et al., 2010) from a vector harboring, in its 5′- and 3′-UTR, RNA sequences derived from *Arc* UTRs (SA-Ch, brief for SynActive-ChR2; Figure 1D). These 5′- and 3′-UTR sequences mediate the translation of the SA-Ch actuator and reporter at potentiated synapses. ChR2 was fused to the red fluorescent protein (RFP) mCherry and to a bi-partite tag at the C-terminus derived from the NMDAR C-terminus consensus sequence (Kornau et al., 1995; Gradinaru et al., 2007) that interacts with PSD components to localize the protein to the postsynaptic domain.

Under resting conditions, SA-Ch transcript is mainly silent, and only a percentage of synapses express SA-Ch. Translation of SA-Ch is increased by chemical LTP induction and is negligible when potentiation is suppressed when NMDAR signaling is blocked with AP5. LTP induction with glutamate uncaging causes the selective expression of SA-Ch at the stimulated synapses, which is blocked by anisomycin application. When expressed in the hippocampus, spines were labeled by SA-Ch after animals explored a new environment. SA-Ch retains the basic properties of the encoded ChR2 variant (Gunaydin et al., 2010), and blue-light illumination is sufficient to drive synaptic events that are not dependent on presynaptic activity, as measured by light-evoked calcium transients. Light-activated SA-Ch mimics the physiology of synaptic activity, since synaptic, but not dendritic, illumination causes local calcium transients, and optogenetic stimulation initiates αCaMKII phosphorylation and accumulation at SA-Ch^+^ synapses (Lisman et al., 2012).

### Significance of AS and SA Reporters

The AS-PARac1 and SA-Ch strategies are based on local translation at synapses undergoing LTP. Translation in dendrites is necessary for most L-LTP forms, and the local application of protein synthesis inhibitors to dendritic regions decreases L-LTP amplitude (Bradshaw et al., 2003). Moreover, the application of the inhibitor of protein synthesis emetine to the apical dendrites of hippocampal CA1 pyramidal neurons impairs L-LTP at apical but not basal dendrites. Both approaches rely on PSD-interacting moieties to localize the newly synthesized protein to the synapse and prevent its diffusion, exploiting what is likely to be a general mechanism for new proteins incorporation during the structural reorganization of synapses undergoing potentiation (Bosch et al., 2014).

The responsiveness to neuron activation is due to *Arc* UTRs, which regulate localization and expression (Pinkstaff et al., 2001; Kobayashi et al., 2005). *Arc* is rapidly expressed after neuron and synaptic activation by electroconvulsive seizures and PP high-frequency stimulation that induce LTP; the administration of the NMDAR blocker MK-801 blocks both LTP and *Arc* expression induction (Lyford et al., 1995). Novel *Arc* synthesis is necessary for induction and consolidation of LTP (Plath et al., 2006), and genetic or antisense *Arc* knock-out in mice and rats impairs L-LTP consolidation (Guzowski et al., 2000). Although ARC protein has been ascribed a complementary role in LTD and homeostatic scaling, *in vivo* stimulations at frequencies that induce LTD (e.g., 1 Hz) do not initiate *Arc* expression (Steward and Worley, 2001), and LTD induced by NMDA-only application does not induce *Arc* and does not require ARC protein (Bramham et al., 2010). Furthermore, acute *Arc* overexpression *in vivo* does not induce LTD (Bramham et al., 2010; Yilmaz-Rastoder et al., 2011); rather, ARC may have a priming effect on synapses (Jakkamsetti et al., 2013). A possibility is that, following LTP induction, ARC protein acts locally to mobilize AMPAR for L-LTP consolidation (Hanley, 2014), thus explaining its accumulation in inactive synapses following LTP-inducing BDNF administration (Okuno et al., 2012). In line with this interpretation, shRNA *Arc* knockdown increases the spreading of SEP-tagged AMPAR to spines surrounding synapses undergoing structural LTP (El-Boustani et al., 2018).

Both AS-PaRac1 and SA-Ch are synthesized following focal LTP protocols, and their expression positively correlates with SEP-GluA1 accumulation and spine dimension (both mRFP and mTurquoise fluorophore have been used in place of the Venus moiety; Hayashi-Takagi et al., 2015; Gobbo et al., 2017). Notably, a proportion of SEP-GluA1 containing synapses does not express SA-Ch, suggesting that in this population of synapses, the threshold for long-lasting potentiation was not reached; consistently, in this group, SEP-GluA1 intensity levels were lower than in the SA-Ch^+^ group (Gobbo et al., 2017). Thus, the tagged population of synapses is the ensemble of synapses undergoing translation dependent long-term potentiation, consistently with the *in vivo* reports. In particular, Hayashi-Takagi et al. (2015) found that following the optogenetic ablation of the synapses tagged in the motor cortex during the learning of a motor task severely impaired the performance of the animals.

### Technical Considerations

The AS-PARac1 and SA-Ch reporters provide both live and remote access to the population of potentiated synapses during the expression of the corresponding transcripts, making them suitable for imaging in living animals (taking into account the protein synthesis time and the maturation time of the fluorophore in the fluorescent protein of the reporter) and in fixed brains. AS-PaRac1 relies to ongoing degradation conferred by the PSDΔ1,2 component that also reduces the half-life of the reporter to 1 or 2 days, making it possible to conduct longitudinal imaging studies even with a constitutive promoter (Hayashi-Takagi et al., 2015).

SA-Ch appears to rely more heavily on translation regulation, conferred by the presence of a larger portion of the *Arc* 3′-UTR and, particularly, of the *Arc* 5′-UTR, which uses a non-canonical, IRES-dependent mechanism of translation (Gobbo et al., 2017). Indeed, Hayashi-Takagi et al. (2015) also observed increased specificity when using the synthetic SARE promoter (Kawashima et al., 2013) rather than the constitutive CAG promoter; indeed, the use of SARE promoter results in the incorporation of part of the *Arc* 5′-UTR in the final AS-PARac1 transcript. Nevertheless, to increase the time resolution to tag and identify potentiated synapses, the use of inducible systems can help, as done for activity tagging of neurons (Reijmers et al., 2007; Kawashima et al., 2014). For example, Hayashi-Takagi et al. (2015) used a synthetic activity-dependent promoter, whereas Gobbo et al. (2017) used a doxycycline-sensitive promoter to initiate the production of the SA-Ch transcript, hence defining the time window for tagging by means of the drug administration.

Although originally developed to express effectors at potentiated synapses, the AS-PARac1 and SA-Ch systems seem flexible enough to express different proteins by changing the cDNA sequence that will be translated into protein. Indeed, the two groups have shown that at least to some extent, the reporters can be modified and transformed into mapping instruments, removing the active component (i.e., Rac1 or the ChR2 moieties) and changing the fluorescent protein to solve spectral issues when imaging (Hayashi-Takagi et al., 2015; Gobbo et al., 2017). For instance, the SynActive vectors, which use the expression cassette of SA-Ch, are being used to drive the expression of proteomic bait reporters, to study the molecular composition of potentiated spines (Mainardi et al., 2018).

### Other Reporters

Other groups have developed reporters for local dendritic translations; however, their focus has mainly relied on gaining insight into the regulation of the expression of particular genes or the effect of specific RNA sequences (Aakalu et al., 2001; Butko et al., 2012; Ifrim et al., 2015). For those interested in a comprehensive evaluation of these reporters, we refer to other excellent reviews (Biever et al., 2019; Holt et al., 2019).

## Synaptic Connectivity

Multiple inputs converge onto the same postsynaptic neurons, and identifying which connections are active during experiences and learning can provide crucial information about the flux of information. This is particularly important when the circuit is not entirely described, or when relevant connections represent only one of many possibilities of pairwise links (as for example in the hippocampal formation). One possibility is to image live synaptic activity of both pre- and postsynaptic neurons, e.g., with GECIs of different colors. For example, Inoue et al. (2019) expressed the red fluorescent XCaMP-R in layer 5 pyramidal neurons and the yellow XCaMP-Y in somatostatin interneurons to detect concomitant synaptic activity in the barrel cortex. This could be potentially extended to synaptic connections between various neurons, either different neuron types or neurons whose soma is located in different brain areas. This could be potentially restricted between engram cell populations forming monosynaptic connections (e.g., CA1-subiculum or PFC-BLA connections) by expressing spectrally distinct GECIs in the two populations with activity tagging technologies.

This conceptual approach has been undertaken by Choi et al. (2018) to visualize connections between CA3 and CA1 engram cells. They adapted the mGRASP technology, which was designed to map synaptic connections between neurons by means of split GFP complementation: the two halves (pre- and post-GRASP) are exposed on the surface of the pre- and postsynaptic neurons separately by fusing it to a transmembrane domain. Only at synaptic interfaces the two halves are at the right distance to reconstruct the full, fluorescent, GFP protein (Kim et al., 2011). Choi et al. (2018) derived cyan and yellow pre-mGRASP variants and expressed them in CA3 constitutively (cyan) from the cfos/tTA activity tagging system (yellow; Garner et al., 2012). Similarly, they expressed the post-mGRASP in CA1 neurons, along with a RFP from a constitutive promoter and a far-RFP in an activity dependent way. Thus, connections between CA3 and CA1 engram cells, as well as the other combinations, can be identified as yellow synapses on far-red CA1 neurons. This enabled the authors to measure morphological and physiological properties of this set of synapses, demonstrating that synapses between CA3 and CA1 engram cells display several potentiation hallmarks. The work by Choi et al. (2018) was actually preceded by Macpherson et al. (2015), who developed a three color variant of the GRASP system. Here, splGFP(1–10; corresponding to pre-GRASP) is fused to synaptobrevin, a protein localized to presynaptic vesicles; hence, when neuronal activity causes the fusion of neurotransmitter vesicles, spl-GFP(1–10) is exposed on the presynaptic membrane. This causes an increase in fluorescence when postsynaptic partners meet, and the activation of multiple pathways has been studied to the *Drosophila* olfactory and thermosensory system using different color variants of this system (Macpherson et al., 2015).

Multiple methods can in principle be combined to gather information about the synapse status and the relative connectivity. In its simplest form, the postsynaptic markers of activity described in the previous sections can be expressed in one region or neuron population, and axons or terminals of different neurons can be labeled expressing a spectrally distinct fluorescent protein. For example, a red AS-PARac1 variant was expressed along with a GFP filler and a blue presynaptic marker (VAMP2-Turquoise) to mark reciprocal connections within the same populations of cortical neurons (Hoshiba et al., 2017). Even if in this proof-of-principle all of the constructs are expressed in the same neurons, it also seems straightforward to express the two transgenes to different pre- and postsynaptic populations. A recent technique named SYNPLA can also be useful to inquire connections between neuronal populations. SYNPLA detects recently activated GluA1-positive synapses by fusing the myc peptide to the extracellular side of presynaptic neurexin; using a combination of oligonucleotide-bound antibodies, rolling circle amplification, and fluorescent nucleotides, it detects juxtaposed myc-neurexin and newly exposed GluA1 as very bright spots (Dore et al., 2020).

### Technical Considerations

Activity-dependent synaptic tracing methods are new technologies that have the potential to provide insight into the activity and plasticity of connections between brain areas as well as local circuits. Of course, when coupling reporters of postsynaptic activity with projection tracers, the same technical considerations hold as if they were used alone. In addition, the combination of techniques calls for a careful evaluation of the imaging and detection conditions, as the crowding of the spectral window increases with the number of different fluorophores; realistically, information about synaptic connectivity in an activity-dependent manner would require three or four different fluorophores. While this is technically feasible, care is advised in order to reduce spectral crosstalk, especially if weak signal is involved, or significant differences in signal intensity are at play. In some cases, the use of reporter variants optimized for orthogonal and high signal-to-background ratio when immunodetected could provide significant advantages (Viswanathan et al., 2015; Gao et al., 2019). Particular care during imaging and image reconstruction would be needed to ensure that optical aberrations are minimized, which could otherwise result in images misalignment and potential identification errors.

## Synapse Clusters

In the previous sections, we have summarized the various approaches that can be used to identify ensembles of synapses involved in particular neural representations. The degree of convergence of the various methods is unclear, similarly to what also happens with cellular tagging (Guenthner et al., 2013; Ramirez et al., 2013; Wang et al., 2017). Nevertheless, a degree of overlap between active synapses, synapses exposing new AMPA receptors, and synapses identified by AS-PARac1 and SA-Ch translation reporters is expected, according to the general understanding of the mechanisms of potentiation (Bliss et al., 2018). For example, both AS-PARac1 and SA-Ch expression correlates with SEP-GluA1 exposure in most synapses.

**Figure 2:**
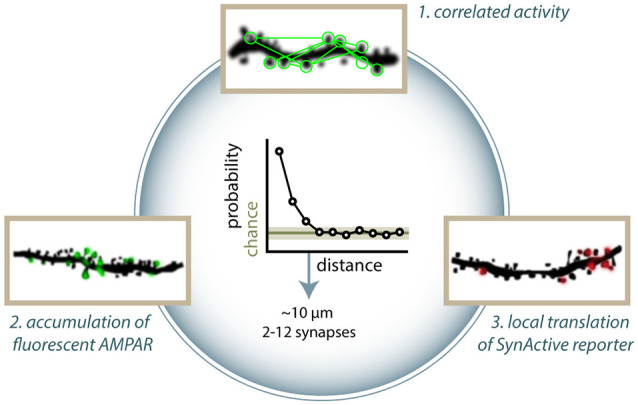
Clustering of active and potentiated synapses. Different approaches have identified distance-dependent correlated activity. (1) Takahashi et al. (2012) showed that groups of co-active synapses can be identified by means of calcium imaging. Makino and Malinow (2011) found that the enrichment of SEP-GluA1 accumulation at synapses is correlated to that of nearby synapses (arrowheads in figure). (3) Similarly, Gobbo et al. (2017) reported that the distance between two potentiated synapses is shorter than what would be expected by chance based on the expression of SA-Ch. Clusters typically range between 2 and 13 synapses. All methods estimate typical cluster dimensions in general agreement to each other, finding that two synapses have a higher than chance probability of have a common active or potentiated status under about 10 μm. Above this threshold, correlation is at chance level. This translates into a cluster dimension of around 2–12 spines.

Notably, many groups have independently reported spatial clustering of synaptic activity, regardless of the detection methods used. Kleindienst et al. (2011) have reported clustering of coincident activity of synapses on CA3 dendrites in cultured slices, with higher co-activation likelihood at intersynaptic distances smaller than 16 μm. Similarly, Takahashi et al. (2012) found clusters of 2–12 co-active synapses performing calcium imaging in CA1 neurons. *In vivo*, clusters of co-active synapses have been detected in the layer 2/3 pyramidal neuron of the somatosensory cortex (Takahashi et al., 2012) and in layer 2/3 and 5 of the visual cortex (Winnubst et al., 2015; Gökçe et al., 2016) with similar distances. In an analogous way, clustering among synapses incorporating GFP-tagged GluA1 has been shown in the barrel cortex (Makino and Malinow, 2011) and in the hippocampus CA1, with typical cluster dimensions around 8 μm (Takahashi et al., 2012). Observing SA-Ch expression, Gobbo et al. (2017) demonstrated preferential clustering of potentiated synapses in both the dentate gyrus and CA1 hippocampal region, with clusters typically containing 2–14 synapses. Such consistency, in terms of number of synapses involved and of intersynaptic distance, is likely to underlie some mutual dependency of the clustered synapses (Figure 2).

Synaptic capture (STC) is a cellular mechanism whereby a weak stimulation, which normally would lead to E-LTP, results in L-LTP if a strong stimulation is applied, within an hour, to a different synapse onto the same pool of neurons. STC is thought to be due, in part, to the generation of diffusible signals (e.g., proteins) from the synapses receiving the strong stimulation. Consistently with the ranges reported above, 10 μm is the threshold distance found for a subthreshold stimulation to induce potentiation when paired to strong stimulation of another synapse at a given position (Harvey and Svoboda, 2007), and NMDAR inhibition with AP5 suppresses clustered co-activity (Takahashi et al., 2012). Similarly, another group found that STC can take place within 30 μm of the tetanized synapse, dropping to zero when the distance between the two spines is 70 μm (Govindarajan et al., 2006). This suggests that clusters of potentiated synapses are linked to mechanisms of synaptic cooperativity. Nonlinear properties of neuronal activity, diffusion of intracellular effectors, or anatomical clustering of inputs could explain this effect (Kastellakis et al., 2015). Regardless of the mechanism, clustering can have a profound impact on memory encoding (Mikhaylova and Kreutz, 2018), facilitating the response to a given stimulus or the encoding of a unitary piece of representation from converging inputs (Winnubst and Lohmann, 2012; Kastellakis et al., 2015). Clustering of potentiated inputs could be a mechanism employed by neurons to store information and facilitate recall, with the additional property of being more robust toward the loss of synapses and transmission noise. Indeed, it has been shown that learning facilitates the formation of new spines in clusters in the retrosplenial cortex (Frank et al., 2018), and LTP induction extends significantly the lifetime of synapses within 5 μm from a potentiated synapse (Wiegert et al., 2018).

## Inhibitory Synapses

The wary reader will have noticed by now that all the reporters we have described so far identify glutamatergic synapses, which almost invariably correspond to particular structures of the postsynaptic neuron, the spines (Holtmaat and Svoboda, 2009). Nonetheless, inhibitory neurons constitute a small (10–15%) but significant proportion of neurons in the brain (Tremblay et al., 2016) and play a fundamental role in information processing in the brain (Müllner et al., 2015). Inhibitory transmission onto excitatory neurons can undergo activity-dependent plasticity (Fagiolini et al., 1994; Hartman et al., 2006; Chiu et al., 2019), which can shape excitatory transmission (Lin et al., 2008; Chevaleyre and Piskorowski, 2014). Inhibitory neurons may even establish complementary ensembles of neurons modulating the activity of ensembles of pyramidal cellular engrams, which may have a compensatory effect to restore the excitation/inhibition balance, contribute to stimulus filtering (for example, in the representation of familiar situations), or act in synergy with principal (excitatory) engrams in context discrimination (Barron et al., 2017). However, studies focused on imaging inhibitory synapses plasticity have been limited for a number of reasons.

First, GABAergic synapses are harder to identify than glutamatergic ones from the morphological point of view, as there is no obvious structural correlate analogous to excitatory synapses, and inhibitory synapses are identified both on dendritic shafts and on the sides of spines, which also contain excitatory synapses (Chen et al., 2012). Hence, inhibitory plasticity can generally be inferred only by the remodeling of the axonal bouton (Wierenga et al., 2008; Keck et al., 2011). An alternative consists in the identification of inhibitory synapses with fluorescent proteins fused to gephyrin, a postsynaptic scaffold protein found at inhibitory synapses (Triller et al., 1985). With Teal-gephyrin, a cyan version of gephyrin, Chen et al. (2012) were able to describe the dynamics of the plasticity of inhibitory synapses in layer 2/3 pyramidal neurons in the visual cortex across days, showing that spine-associated inhibitory synapses are more dynamic than those located on the dendritic shafts. Furthermore, visual experience induces a spatially clustered reorganization of inhibitory synapses in correspondence to dynamic spines, suggesting that inhibitory and excitatory plasticity are correlated (Chen et al., 2011; Villa et al., 2016).

Second, inhibitory plasticity is less characterized than excitatory synapses (Kano, 1995; Castillo et al., 2011; Flores and Méndez, 2014), not least because of the plethora of plasticity forms (Maffei, 2011; Chiu et al., 2019) and of diverse interneuron populations (Kubota et al., 2016; Tremblay et al., 2016; Pelkey et al., 2017). In any case, it is difficult to image inhibitory activity and plasticity. Inhibitory activity can be inferred by recording the activity of presynaptic boutons with GECIs (Inoue et al., 2019). Theoretically, inhibition could be imaged with voltage sensors (Canepari et al., 2010) or with chloride sensors to identify the flux of Cl^−^ ions from GABA_A_ channels. However, the two latest available chloride sensors, Chlomeleon (Grimley et al., 2013) and Chlophensor (Sato et al., 2017), are generally used to image whole neuron chloride dynamics, and they have not been shown to be usable at synaptic resolution. In addition, inhibitory synaptic transmission does not always result in chloride influx and could act in the form of shunting inhibition. Lastly, the recent iGABASnFR, analogous to the glutamate sensor iGluSnFR, signals gamma aminobutyric acid (GABA) binding with an increase in green fluorescence (Marvin et al., 2019). Starting from candidate GABA-binding proteins identified with genomic mining in the bacteria gene pool, the authors inserted cpEGFP in their sequences and came up with a few candidate GABA sensors. Although it might constitute a solid stepping stone, iGABASnFR still does not appear powerful enough to image inhibitory activity at the single-synapse level.

## Extending the Toolbox

### Neuromodulators

Besides glutamate and GABA, other neurotransmitters and neuromodulators may have profound effects on neural activity and information processing. Furthermore, neuromodulators can modify the plasticity properties of synapses, e.g., by lowering the threshold to produce potentiation at synapses (Takeuchi et al., 2016). In recent years, genetic sensors for modulators, such as dopamine, norepinephrine, and others, have been reported. While they have been shown to detect neuromodulators release *in vivo*, their relatively low fluorescence has so far limited their use to whole-cell applications (Leopold et al., 2019). Hence, they do not seem to detect the release of neuromodulators at single synapses or release terminals. Nonetheless, continuous improvements of the most promising variants could produce in the near future sensors able to detect the release of dopamine and other modulators with single-synapse resolution.

### Inhibitory Neurons

Most of the imaging applications of synaptic activity have been conducted in excitatory neurons, either pyramidal neurons in the cortex and the hippocampus or granule cells in the dentate gyrus. Notably, inhibitory neurons undergo synaptic plasticity too (Chen et al., 2013; He et al., 2016; Feese et al., 2018), and sensory activity shapes the plasticity of neural inhibition (Fagiolini et al., 1994). While calcium imaging of synaptic transmission has been successfully performed also in inhibitory neurons (Chen et al., 2013), it is unclear how the reporters for synaptic potentiation would perform in GABAergic neurons, also considering that a great percentage of these neurons do not display spines (Scheuss and Bonhoeffer, 2014; Pelkey et al., 2017).

### Other Forms of Plasticity

Potentiation of synaptic transmission is a major topic in memory research, which could explain why the development of tools to visualize synaptic plasticity has been focused on LTP reporters. Indeed, LTP and LTD can bi-directionally modulate both the amplitude of synaptic responses and memory performance (Collingridge et al., 2010; Nabavi et al., 2014). LTD is generally elicited by low-frequency stimulations, although other forms exist, and shares a significant proportion of the molecules involved in LTP, which, along with the fact of being somewhat less characterized than LTP at the molecular level, probably explains the lack of LTD-selective sensors (Kemp and Manahan-Vaughan, 2007). Indeed, some molecules including betaCaMKII and phosphatases, such as PP1, seem to be crucial in establishing LTD and could be helpful in the design of LTD sensors.

Because LTD typically results in the removal of AMPAR from the surface of depotentiated synapses, some of the tools used to monitor potentiation could be used in principle to also detect LTD. Indeed, a SEP-fused AMPA receptor subunit 2 (SEP-GluA2) is internalized in response to LTD induction, even though this has not been tested *in vivo* yet (Ashby et al., 2004). Analogously to LTP, synaptic LTD could be inferred by synaptic shrinkage or elimination using diffusible cell fillers; functional LTD and depotentiation could be inferred using GECIs from a decrease in the amplitude of ΔF/F synaptic events (Wiegert et al., 2018). Reducing synaptic transmission at the olfactory glomeruli in *Drosophila* using temperature-sensitive mutants showed reduced postsynaptic ΔF/F events measured with a postsynaptic localized dHomer-GCaMP3 fusion, although longitudinal changes have not been measured (Pech et al., 2015).

Besides the Hebbian form of plasticity, such as LTP and LTD, other forms of plasticity have been described. For instance, metaplasticity has been described as a mechanism whereby prior plasticity at a particular synapse modifies the response to subsequent stimulations compared with naïve synapses and can assume the form of a change in threshold, amplitude, or even direction of change (increase or decrease in amplitude; Abraham, 2008). Homeostatic plasticity generally occurs on longer timescales and has a net effect of counterbalancing changes in activity that drives the system away from an equilibrium point, e.g., reducing neuron excitability after sustained incoming activity (Turrigiano, 2017). Furthermore, both homosynaptic and heterosynaptic forms of plasticity exist, as well as forms of circuit plasticity. Overall, these mechanisms are less understood at a molecular level and share a significant proportion of the molecules involved in LTP and LTD. Nonetheless, some of the tools to image synaptic activity could find application to these forms of plasticity. Where AMPA receptors insertion or removal from the plasma membrane is involved, SEP-GluA sensors could be used as indicators. Different forms of homeostatic plasticity have been reported to act either by modifying the postsynaptic response to a release of a quantum of neurotransmitter or by changing the probability of released neurotransmitter. Therefore, GECIs and pHluorin-based sensors could find application in monitoring changes in calcium influx at the pre- or postsynaptic terminal or in the rate of vesicle fusion with the presynaptic membrane. Indeed, Pech et al. (2015) employed the presynaptic pH-sensitive synaptophysin-pHTomato and the postsynaptic dHomer-GCaMP3 to demonstrate that prolonged exposure of *Drosophila* flies to apple odor over multiple days causes a reduction in postsynaptic response, but not of presynaptic vesicle fusion, in the olfactory projecting neurons in the antennal lobe.

## Reporters and Effectors in Synaptic Engram Research

As we have discussed in the previous sections, the idea that synaptic engrams underlie the formation and maturation of cellular engrams is still to some extent an untested idea, despite the large amount of correlative evidence linking memory formation and synaptic potentiation (Takeuchi et al., 2014; Nomoto and Inokuchi, 2018). Indeed, the merge of two unitary cell engrams in a behaviorally relevant framework by means of their concomitant optogenetic reactivation is dependent on NMDAR activation and novel protein synthesis (Ohkawa et al., 2015), and anisomycin administration during memory encoding impairs the reactivation of the cellular engram by natural stimuli (Ryan et al., 2015). Indeed, spatial memories can also be formed in the absence of neural activity, but are dependent on NMDAR activity (Rossato et al., 2018). Hence, there is still considerable debate regarding the role of potentiated synapses in the establishment, storage, and recall of memories (Tonegawa et al., 2015b; Poo et al., 2016). While some argue that synapses and their modifications may be too short lived to provide a reliable substrate for memory (Mongillo et al., 2017), synaptic engrams could still support the representation of memories on shorter-than-lifetime timescales. Indeed, the half-life of hippocampal synapses has been reported to be surprisingly short (Attardo et al., 2015), but this could mirror the fact that the remote memories are less sensitive to hippocampal lesion or inactivation than recent memories (Squire, 1986; Squire et al., 2015; Kitamura et al., 2017). Cortical synapses have a significantly longer half-life (Yang et al., 2009; Attardo et al., 2015), and synapses tend to be more stable and last longer than other connections (Wiegert et al., 2018). In addition, the redundancy of synaptic engrams could make them robust toward noise in activity and loss of synapses, and multiple events of reconsolidation could support both the maintenance and flexibility of synaptic engrams, analogously to what happens with memories (Dudai, 2011).

Similar controversies about the existence of cellular engrams were solved with the development of new molecular tagging techniques (Eichenbaum, 2016). Given the properties of memory and potentiation, there is no need to exclude *a priori* a role of synaptic engrams in the formation and maturation of cellular engrams or even an interplay between synaptic and cellular engrams (Dudai and Morris, 2013). We believe that the use of the reporters and effectors we have described could contribute to solve at least some of the questions regarding the relative role of synaptic and cellular engrams. For instance, describing the relative allocation of active synapses and potentiated synapses between engram and non-engram cells could provide meaningful insight into the interplay between synaptic and cellular activity in memory paradigms. For example, it has been shown that the connections between CA3 and CA1 engram cells have a higher overall amplitude, and electrical LTP is occluded at these synapses, suggesting that potentiation had occurred at these synapses (Choi et al., 2018).

### Disrupting and Recalling Activity at Potentiated Synapses

**Figure 3:**
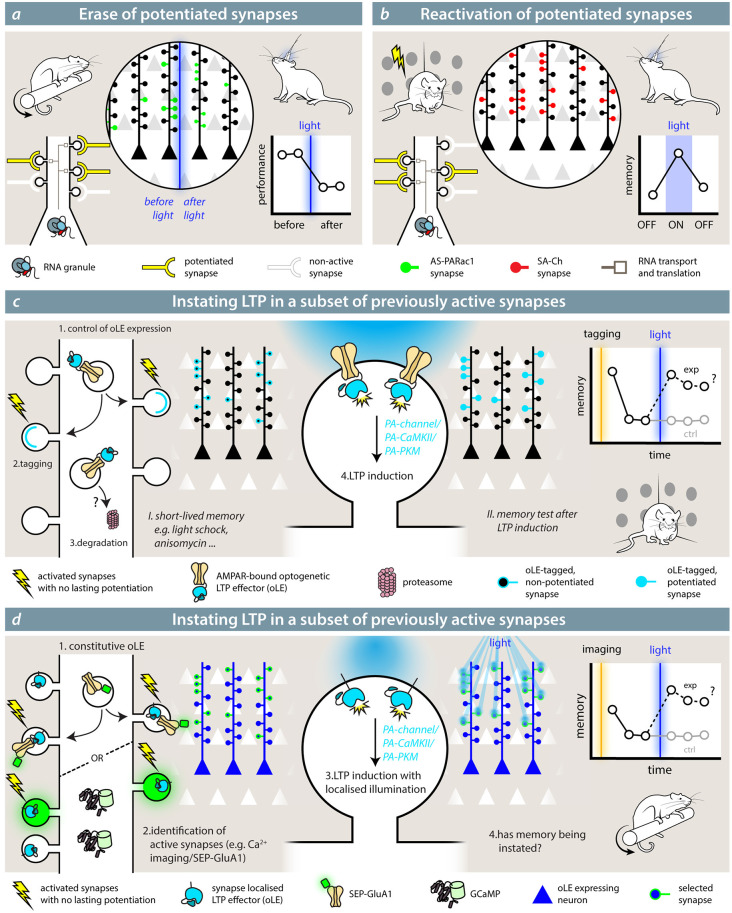
Synaptic effectors in the study of memory. **(A)** Hayashi-Takagi et al. (2015) used the optogenetic reporter for synaptic potentiation AS-PARac1 to erase potentiated synapses by the learning of a motor memory task (rotarod). After learning, synapses express AS-PARac1. After blue-light illumination, labeled spines are shrunken, and the performance of the animal in the rotarod task is significantly reduced. **(B)** With SA-Ch (or any SA-Ch* opsin variant; Gobbo et al., 2017), it is possible to reactivate synapses that underwent potentiation during the learning (tagging) phase. If these play a role in the representation of the associated memory, re-activating them would elicit a coherent behavior during light illumination. While experiments outlined in **(A,B)** demonstrate the necessity role of potentiated synapses in the representation of a memory, theoretical experiments **(C,D)** are mimicry experiments aimed at demonstrating that potentiation of a set of synapses is sufficient for the formation and expression of a memory. The optogenetic LTP effector (oLE) could be based on either kinases that have been shown to have a prominent role in LTP, i.e., PKMζ or a constitutively active form of αCaMKII or light-sensitive NMDAR channels. To control their activity, a light-sensitive form of the two kinases has to be devised. The animal is first trained under conditions that do not form a long-lasting memory and/or impede potentiation; for example, a weak training, such as mild-shock contextual fear conditioning, anisomycin infusion, etc. In **(C)**, the oLE localization to relevant synapses is achieved by fusing it to a GluA1 subunit, and it is coupled to AMPAR exposure. Control of expression would be critical, but specificity could be improved by increased degradation. If the interpretation of the role of LTP is correct, induction of LTP at this set of synapses would cause the formation of a memory. In **(D)**, the opto-LTP effector is present at all synapses. The experimenter first detects active synapses by means of an imaging reporter (calcium imaging or visualization of SEP-GluA1, for example), then selectively activates the oLE at the selected synapses with patterned illumination.

Testing the role of potentiated synapses in loss- or gain-of-function experiments could be performed by inhibiting or recalling the activity of potentiated synapses. If a given ensemble of synapses potentiated during a learning paradigm is relevant for the representation and storage of its memory, manipulating the activity of this synaptic ensemble should have a coherent effect on memory recall. Hayashi-Takagi et al. (2015) showed that a task-specific representation can occur at the synaptic level. Using the catalytic properties of AS-PARac1, they first trained the animal in a motor task (rotarod), then they optogenetically wiped out the potentiated synapses expressing AS-PARac1. As a result, the motor memory the animals had acquired was *de facto* erased (Figure 3A). Importantly, the performance in a previously learned different task was unaffected, demonstrating the specificity of the synaptic representation of the motor memory. These findings show that potentiated synapses play a role in the representation of a motor memory in the cortex. AS-PARac1 only provides unidirectional manipulations of synaptic activity, since disrupting the spine structure is not reversible.

Conversely, after tagging potentiated synapses during the learning phase, SA-Ch expression at potentiated synapses could be used to recall their activity at a later stage, thus observing if their reactivation is sufficient to recall the encoded memory (Figure 3B; Gobbo et al., 2017). Although the synaptic events elicited by the optogenetic activation of SA-Ch are smaller in amplitude than the spontaneous ones from neurotransmitter release, this is likely due to the ChR2 variant employed (ChETA), which has rather small photocurrents compared with other ChR2 variants (Mattis et al., 2011). Given that most ChR2 differ from each other by a handful of point mutations, changing the ChETA moiety with a ChR2 with larger photocurrents (Dawydow et al., 2014) should be sufficient to match postsynaptic events, while maintaining the same pattern of expression as SA-Ch. Alternatively, single channel chemogenetic tools could be expressed at potentiated synapse, making it possible to re-excite potentiated synapses in larger brain volumes (Magnus et al., 2011). In principle, then, also inhibitory ChR2 variants or hyperpolarizing ion pumps could be expressed at potentiated synapses to inhibit synaptic activity reversibly (Wiegert et al., 2017).

### Instating Synaptic Plasticity at Synapses

To probe the causal role of potentiation, one would need to artificially induce LTP on a set of synapses activated by a stimulus and observe if this results in the creation of a memory, thus mimicking a learned response to the very stimulus. The difference with the previous experiments (i.e., gain- and loss-of-function alterations of the activity of potentiated synapses) is conceptual, and it is analogue to the difference existing between the tag-and-recall experiments on engram neurons (Liu et al., 2012; Ramirez et al., 2013) and the experiments performed by the Silva and Josselyn groups, where they skewed the probability of a subset of neurons to allocate a memory by CREB overexpression in those neurons (Han et al., 2009; Kim et al., 2014).

Testing the prediction that LTP at a given set of synapses is sufficient for engram formation has been originally proposed with a *Gedankexperiment* by Richard Morris (Takeuchi et al., 2014); how to actually implement it is a different story, and the author themselves breeze through the issue assuming a hypothetical light-sensitive calcium channel equipped with a tagged synapse-targeting sequence. The first, and crucial, step would be to identify or tag the synapses that are active during the presentation of the stimulus. This would need to happen under conditions that do not result in synapse potentiation (e.g., a subthreshold stimulation, a weak behavior paradigm, anisomycin infusion) and do not create a lasting memory. If memory were reinstated after artificially inducing LTP at these synapses, it would provide a definitive demonstration of the causal role of potentiation in memory. The conundrum lies in how to actually implement this with the available (or, at least, plausible) molecular tools.

The optogenetic LTP effector (oLE) would need to act on a signaling cascade that causes synaptic potentiation, but should be normally present—or recruited—at synapses in an inactive form. Theoretical but plausible candidates are constitutively active forms of αCaMKII (Lledo et al., 1995) or PKMζ (Ron et al., 2011; Schuette et al., 2016), whose activity is hindered by a light-sensitive LOV domain, an approach employed by a number of gated enzymes, including PARac1 (Leopold et al., 2018). Light stimulation removes the steric block of the LOV domain, activating the enzyme. Notably, a light-sensible form of PKA already exists (O’Banion et al., 2018), and optogenetic inhibition of αCaMKII activity has already been successfully performed, suggesting that potentiation is necessary for memory formation (Murakoshi et al., 2017). Alternatively, the oLE could be a membrane Ca^2+^ channel: for instance, the LiGluN2A(V713C), a light-gated NMDAR, can induce structural and functional potentiation after single-spine optogenetic activation when supplemented with a photo-switchable azobenzene compound (Berlin et al., 2016).

To restrict its activity to the relevant synapses, the oLE could be fused to the intracellular terminus of SEP-GluA1-like reporters, which are exposed at synapses during E-LTP, either at the C-terminus (LOV-gated enzymes) or as tandem transmembrane protein (light-sensitive NMDAR; Figure 3C). This would ensure that the oLE is only recruited in a subset of synapses along with SEP-GluA1; hence, LTP could be induced selectively with wide field illumination using standard optic fibers. Background could be reduced with inducible expression system or by active degradation of the extrasynaptic pool. Alternatively, specificity can be obtained by restricting the illumination to the desired subset of synapses even if the oLE is present at all postsynaptic sites (Figure 3D). First, relevant synapses are identified by means of calcium imaging or other activity reporters (e.g., SEP-GluA1), and their position annotated. Then, the oLE is activated by means of two-photon or holographic laser stimulation (Reutsky-Gefen et al., 2013). To avoid spectral crosstalk, GECIs with longer wavelength absorption (Inoue et al., 2019) or red pH-sensitive fluorescent proteins (Shen et al., 2014) can be used, since LOV-based optogenetic switches are usually blue-light sensitive. While theoretical, the described oLE would provide great insight into the exact role of synaptic activity and plasticity in memory, extending all-optical read-and-reactivate technologies already available at the whole neuron level (Marshel et al., 2019).

## Conclusions

In this article, we have tried our best to present in a systematic way the available technologies that allow to image synaptic activity and plasticity. This includes reporters to map and identify active and potentiated synapses and proteins that can recall or hinder synaptic activity.

The introduction of new molecular techniques, such as activity tagging of neurons, has caused a small revolution in the field on neuroscience. Similarly, employing techniques to address the role of subcellular compartments could expand our knowledge of brain functions and of the representation of memories. Being able to identify cells that encode a specific memory is undoubtedly one of the great achievements of modern neuroscience, shining light onto century-old questions (Schacter et al., 1978; Josselyn et al., 2017). However, the role of synaptic potentiation still does not fit smoothly in the picture, and the interplay between synaptic plasticity and cellular engrams is still unclear (Poo et al., 2016). This also reflects on the open questions regarding how engram cells wire together to form a unitary ensemble, and how single, unitary ensembles—which could represent different components of a complex memory, e.g., the spatial information, the emotional value, etc.—are merged together to form a unitary overall representation (Morris, 2003; Mayford et al., 2012; Eichenbaum, 2016).

The emergence of recent articles directly addressing the role of synaptic potentiation in the framework of cellular engrams and the plasticity of these cell ensembles (Ryan et al., 2015; Abdou et al., 2018; Rossato et al., 2018; Ghandour et al., 2019) demonstrates the renewed interest in synaptic plasticity. After the “golden era” of LTP (Bliss and Lømo, 1973; Collingridge et al., 1983; Morris et al., 1986; Frey and Morris, 1997), the development of new techniques has enabled a shift in how the study of synaptic activity and potentiation is approached. Matsuzaki et al. (2004) pioneered the employment of imaging to describe synaptic plasticity. In recent years, more and more molecular techniques have been added to the toolbox to image and act on active synapses, with subcellular precision (Lin et al., 2013; Sinnen et al., 2017; Kakegawa et al., 2018). This is likely to expand further in the next future and find application in the study of learning and memory.

## Author Contributions

AC and FG conceived the project, discussed the ideas and wrote the manuscript. FG prepared the figures with input from AC. All authors contributed to the article and approved the submitted version.

## Conflict of Interest

The authors declare that the research was conducted in the absence of any commercial or financial relationships that could be construed as a potential conflict of interest.
